# A systematic review on the effectiveness of dialectical behavior therapy for improving mood symptoms in bipolar disorders

**DOI:** 10.1186/s40345-023-00288-6

**Published:** 2023-02-05

**Authors:** Brett D. M. Jones, Madeha Umer, Mary E. Kittur, Ofer Finkelstein, Siqi Xue, Mikaela K. Dimick, Abigail Ortiz, Benjamin I. Goldstein, Benoit H. Mulsant, Muhammad I. Husain

**Affiliations:** 1 Department of Psychiatry, Temerty Faculty of Medicine, University of Toronto, Toronto, Canada; 2 Centre for Addiction and Mental Health, Toronto, Canada

**Keywords:** Bipolar disorder, DBT, Psychotherapy, Depression

## Abstract

**Background:**

Evidence-based psychotherapies available to treat patients with bipolar disorders (BD) are limited. Dialectical behavior therapy (DBT) may target several common symptoms of BD. We conducted a systematic review on the efficacy of DBT for mood symptoms in patients with BD. The systematic search used key words related to DBT and BD in Medline, Embase, PsycInfo, CINAHL, and Cochrane Library databases from 1980 to April 1st, 2022. We included studies that enrolled patients with a BD I or II diagnosis (DSM or ICD), age 12 and older who received a DBT-based intervention. Studies reviewed were clinical trials including observational studies that reported at least one outcome related to BD mood symptoms or severity. We did not exclude based upon psychiatric or physical co-morbidity.

**Results:**

We screened 848 abstracts and reviewed 28 full texts; 10 publications with 11 studies met our pre-determined eligibility criteria. All but one were feasibility pilot studies and most included participants in all mood states except for mania. The studies provided preliminary evidence suggesting these interventions may be effective for improving several core symptoms of BD. Overall, all the studies consistently supported that DBT-based interventions are feasible and acceptable for patients with BD.

**Conclusion:**

DBT may be an effective treatment for BD; however, the confidence in this conclusion is limited by the small sample sizes, heterogeneity, and high risk of bias in all published trials. Larger well-designed RCTs are now required to establish the effectiveness of DBT in BD.

**Supplementary Information:**

The online version contains supplementary material available at 10.1186/s40345-023-00288-6.

## Introduction

Bipolar disorders (BD) are characterized by a pattern of chronic relapsing and remitting manic, hypomanic, depressive, or mixed episodes, with residual symptoms and inter-episodic mood fluctuations. The cyclical changes in mood episodes causes significant interpersonal strain and contributes to increased morbidity within the BD population (Sewall et al. 2020). When managing their illness, patients with BD typically receive pharmacotherapy. However, despite advances in these pharmacotherapies, remission and recovery rates remain low. In some longitudinal studies, nearly half of patients experienced recurrences despite optimal pharmacotherapy (Perlis et al. 2006). The limitations of pharmacotherapy alone have led to an increasing interest in the development and adaptation of BD–specific psychotherapies (Oud et al. 2016).

Several well-established psychotherapy protocols have been studied in BD, such as family focused therapy (FFT), cognitive behaviour therapy (CBT), psychoeducation, and interpersonal and social rhythm therapy (IPSRT) (Oud et al. 2016). In the large-scale randomized Systematic Treatment Enhancement Program for Bipolar Disorder (STEP-BD) study, all three psychotherapies resulted in similar response rates when combined with pharmacotherapy (Miklowitz et al. 2007). In a recent network meta-analysis of 39 studies, manualized skills-based psychotherapy interventions combined with pharmacotherapy provided the greatest benefit for BD in terms of improved stabilisation and lowered rates of recurrences or attrition, compared to treatment as usual (TAU) (Miklowitz et al. 2021). Psychoeducation has also been shown to reduce recurrence of episodes, length of hospital admission, and enhance adherence to treatment (Rabelo et al. 1407). Despite this evidence supporting their effectiveness in BD, these psychotherapies remain second- and third-line choices in regional and international treatment guidelines and they are relatively under-utilized (Yatham et al. 2018).

Dialectical behavior therapy (DBT) is part of the ‘third wave’ of cognitive therapies, with a modular and hierarchical structure. It was developed for treatment of difficult to treat borderline personality disorder but has been applied to multiple psychiatric disorders (Linehan and Wilks 2015). It consists of individual psychotherapy, group skills training, on-call telephone coaching, and DBT team meetings (Linehan and Wilks 2015). Using a combination of cognitive and behavioral strategies, the goal of DBT is the development of skills that promote mindfulness, interpersonal effectiveness, emotion regulation, and distress tolerance (Dimeff and Linehan 2001). Given the modularity of DBT, specific components can be used for specific clinical populations (Linehan and Wilks 2015).

While DBT was originally developed to treat borderline personality disorder (BPD) (Linehan et al. 1993), some symptoms of BPD and BD overlap, including mood instability, impulsive behaviours, and suicidality, which can be mood state dependent. Given this overlap in some core symptoms and heterogeneity in presentations, clinicians and researchers have proposed that DBT may be beneficial to target some BD symptoms (McMahon et al. 2016). They have theorized that DBT could be applied to help individuals with BD to become more attuned to their mood changes, enhancing symptom awareness, and potentially encouraging earlier support-seeking prior to the exacerbation of a mood episode (Dijk et al. 2013). In this context, we conducted a systematic review of clinical trials assessing the efficacy of DBT in patients with BD.

## Methods

The review was conducted in accordance with the Preferred Reporting Items for Systematic Reviews and Meta-analyses (PRISMA) statement (Shamseer et al. 2015) and registered on PROSPERO (CRD42021293873).

### Eligibility criteria

#### Study design and participants

We reviewed clinical trials that recruited participants of any gender older than 12 years in any mood state with a diagnosis of BD according to the criteria of the International Classification of Diseases (ICD) or the Diagnostic and Statistical Manual of Mental Disorders (DSM) (Association 2013; WHO 2018). Reviewed trials could include participants with psychiatric or physical comorbidities. While we reviewed clinical trials, including observational trials, only RCTs and cluster RCTs were considered for a meta-analysis.

#### Interventions and comparators

We reviewed trials of psychotherapeutic interventions based on DBT, including adaptations that do not utilize all the components of DBT (i.e., DBT skills training (DBT-ST), individual psychotherapy, coaching, case management strategies, DBT team meetings). DBT-ST interventions were classified based on their components (i.e., at least one of: mindfulness, emotion regulation, distress tolerance, or interpersonal effectiveness skill). However, studies of other established therapies that incorporate one component of DBT (e.g., mindfulness cognitive behaviour therapy—MCBT) were excluded. Studies of DBT interventions meeting the above criteria were included regardless of whether they used a comparator or the nature of the comparator.

#### Outcome measures

We included only trials that reported at least one outcome related to: recurrence rate, severity of symptoms in any polarity, suicidality, substance use, or psychosocial functioning. We included any validated scales related to these symptoms. The primary outcome was effectiveness as measured by core mood symptoms using any validated tool, e.g., the Hamilton Rating Scale for Depression (HDRS) (Hamilton 1960), Beck Depression Inventory (BDI) (Beck et al. 1961), Montgomery–Asberg Depression Rating Scale (MADRS) (Montgomery and Åsberg 1979), or Young Mania Rating Scale (YMRS) (Young et al. 1978). When several scales were used, the primary outcome included in analyses was selected hierarchically, with clinician-rated scales given priority over self-rating scales. Secondary outcomes included scales other than those assessing other mood symptoms, e.g., Distress Tolerance Scale (DTS) (Simons and Gaher 2005), the Mindful Attention Awareness Scale (MAAS) (Brown and Ryan 2003) or qualitative data (e.g. feasibility and acceptability) as reported in the primary studies. We also reported feasibility and acceptability as a secondary outcome.

#### Language and time frame

We included only publications in English between 1980 and April 1, 2022 because DBT was developed in the mid- to late 1980s.

### Information sources and search strategy

The following databases were searched: Medline, Embase, PsycInfo, CINAHL, and Cochrane Library. Additionally, the reference lists of all identified articles were reviewed for other potentially relevant studies. We used the thesauri of each relevant database (e.g., MeSH), using both index and free text terms relevant to the Participant and Intervention sections. The strategy was modified for each individual database search. The keywords used were: Bipolar Disorder, Mania, Hypomania, Bipolar Depression, Dialectical Behavior Therapy, DBT, mindfulness, distress tolerance, emotion regulation, and interpersonal effectiveness skills. The full OVID (MEDLINE, Embase, PsychInfo) search strategy is included in the supplement. The CINAHL and Cochrane searches were based off the OVID search.

### Study selection

Based on the predefined eligibility criteria, the titles and abstracts of all studies identified by the systematic search were screened independently by two reviewers (BDMJ & MU). These reviewers obtained and assessed the full text reports for the studies they deemed to meet the eligibility criteria. Disagreements were resolved through consultation with a third reviewer (MIH).

### Data extraction and data items

Data extraction was conducted independently by two reviewers (MEK, MU) using a standardized data extraction form developed by the research team. The form includes: a description of the study sample (N, mean age, gender, diagnosis); the therapeutic intervention (e.g., DBT or DBT-ST; comparator group; trial design; duration of follow-up; outcomes; and publication year and status of reports. When effect sizes could not be calculated, we contacted the authors to obtain these effect sizes, or the data needed to calculate them.

### Risk of bias assessment

The quality of the included studies was measured by two reviewers (BDMJ, MEK) using the Cochrane risk of bias tool (Higgins et al. 2011), which assesses five domains: selection (randomization and allocation concealment), performance (blinding of participants and research personnel), attrition (incomplete outcome data), detection (blinding of outcome assessment), and reporting (selective outcome reporting). We classified the risk of bias as: low risk if there were some concerns of bias for none of the five domains or at most one of them); unclear risk of bias (if these were some concerns of bias for two or three of the five domains); or high risk of bias (if these were some concerns of bias for four or five domains and at last one domain with a high risk of bias).

### Protocol deviation

Given the high risk of bias and the heterogeneity of the interventions, outcomes, and participants, we did not perform a meta-analysis.

## Results

**Fig. 1:**
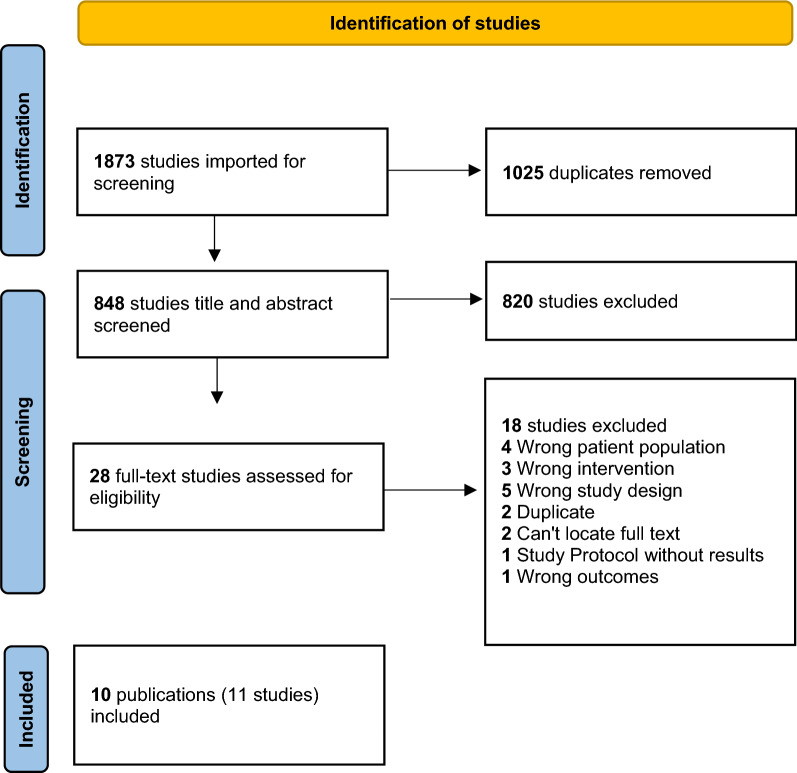
PRSIMA flow diagram

The results of the search are summarized in Fig. 1: 848 unique abstracts were screened; 28 full texts were reviewed; and 10 publications reporting on 11 studies met all eligibility criteria and were included in the final review. Of these 11 studies, six were RCTs and five were observational studies. Table 1 summarizes the characteristics of the 11 included studies and Table 2 summarizes their effectiveness outcomes. Additional file 1: Table S2 in the supplementary material describes the included interventions and reported feasibility and acceptability measures.

**Table 1:** Descriptive characteristics for included study samples

Authors, year	Study design	Diagnosis	Mood state		Symptom scales	Intervention group			Control group		
			Included	Excluded		N (enrolled)	Sex (M/F)	Age	N (enrolled)	Sex (M/F)	Age
Goldstein et al. 2007	Single arm *(Pilot open trial)*	BD1: 7 BD2: 2 NOS: 1	–	Manic, depressed, mixed (past 3 months)	K-SADS-DRS K-SADS-MRS	10	2/8	15.8 (1.5)	–	–	–
Goldstein et al. 2015	RCT *(Pilot, unblinded, 2:1)*	BD1: 3 BD2: 8 NOS: 9	–	Manic depressed, mixed (past 3 months)	K-SADS-DRS K-SADS-MRS	14	3/11	15.82 (2.1)	6	2/4	16.83 (1.4)
VanDijk et al. 2013 *Main study*	RCT *(Pilot)*	BD1: 10 BD2: 14	Euthymic, depressed, hypomanic	–	BDI-II	12	3/9	40.2 (12.8)	12	3/9	41.6 (7.1)
*Secondary analysis*	Single arm	–	–	–	BDI-II	75	–	–	–	–	–
Afshari et al. 2020	RCT *(Pilot, open)*	BD1: 18 BD2: 42	N.R	Manic	BDI-II YMRS	30	14/16	36.00 (6.00)	30	12/18	37.00 (6.00)
Zargar et al. 2019	RCT	BD1: 49	Maintenance phase	Manic	BDI-II YMRS	24	11/13	32.25 (9.57)	25	9/16	29.68 (5.12)
Wright et al. 2020	Single arm *(Feasibility study, open)*	BD1: 5 BD2: 5 NOS: 2	–	Manic	BDI-II PHQ-9 BMRS ASRM	12	3/9	42 (11)	-	-	-
Wright et al. 2021	RCT *(Feasibility study, 2 sites)*	BD1: 37 BD2: 6	–	Manic, depressed	BMRS PHQ-9 HDRS	22	8/14	43.6 (13.0)	21	12/9	49.3 (14.5)
Valls et al. 2021	RCT *(Parallel two-armed, rater-blind)*	BD1: 41 BD2: 24	Euthymic	–	HDRS YMRS	28	16/12	47.61 (7.34)	37	15/22	45.81 (9.92)
Eisner et al. 2017	Single arm *(Open proof of concept pilot)*	BD1: 37	–	Manic, depressed, mixed	HDRS YMRS	37	10/27	41.3 (11.2)	–	–	–
Painter et al. 2019	Single arm *(Proof of concept open trial)*	BD1: 16	–	2 + manic/depressed symptoms	YMRS HDRS	16	8/8	43.19 (12.76)	–	–	–

*BD* bipolar disorder, *NOS* bipolar disorder not otherwise specified,—not reported, *K-SADS-(DRS/MRS)* kiddie schedule for affective disorders and schizophrenia—(depression rating scale/mania rating scale), *RCT* randomized controlled trial, *BDI-II* beck depression inventory, *YMRS* Young Mania Rating Scale, *PHQ-9* patient health questionnaire 9-item, *BMRS* bech mania rating Scale, *ASRM* altman scale for rating mania, *HDRS* hamilton depression rating scale

**Table 2:** Pre to post-treatment means on key outcome measures

Study, authors	Study arm	Depression		Mania		Suicidality		Emotion regulation		Psychosocial Functioning	
		Pre	Post	Pre	Post	Pre	Post	Pre	Post	Pre	Post
Goldstein et al. 2007	INT	K-SADS DRS: 23.1 (12.0)*	K-SADS-DRS: 14.9 (10.6)*	K-SADS MRS: 13.3 (12.1)	K-SADS MRS: 12.2 (14.2)	MSSI: 6.7*	MSSI: 0*	CALS-C: 36.1 (19.47)* CALS-P: 35.5 (17.37)*	CALS-C: 17.78 (24.24)* CALS-P: 27.88 (23.2)*	MESSY: N.R	MESSY: N.R
Goldstein et al. 2015	INT	K-SADS DRS: 25.71 (7.1)*	K-SADS DRS: 9.44 (9.14)*/**	K-SADS MRS: 27.14 (8.5)*	K-SADS MRS: 11.67 (7.89)*	SIQ-JR: 19.17 (21.0)	SIQ-JR: 4.29 (7.67)	CALS-C: 40.17 (19.1)* CALS-P: 41.79 (15.8)*	CALS-C: 15.71 (11.32)* CALS-P: 17.86 (12.95)*	–	–
	CTL	K-SADS DRS: 19.17 (9.6)	K-SADS DRS: 16.5 (7.78)**	K-SADS MRS: 14.50 (6.7)	K-SADS MRS: 11.67 (7.89)	SIQ-JR: 15.50 (29.7)	SIQ-JR: 8 (9.9)	CALS-C: 31.00 (18) CALS-P: 21 (14.3)	CALS-C:37.5 (34.65) CALS-P: 30 (N.R.)	–	–
Van Dijk et al. 2013 *Main study*	INT	BDI-II: 24.6 (8.4)*	BDI-II: 7.9 (6.1)*	–	–	–	–	ACS Total: 4.4 (0.6)	ACS Total: 3.5 (0.6)	–	–
	CTL	BDI-II: 33.7 (11.5)*	BDI-II: 23.58 (17.1)*	–	–	–	–	ACS Total: 4.6 (0.8)	ACS Total: 4.1 (1.5)	–	–
*Secondary analysis*	INT	BDI-II: 25.2 (11.9)*	BDI-II: 16.6 (14.0)*	–	–	–	–	ACS Total: 4.5 (0.9)*	ACS Total: 4.0 (1.2)*	–	–
Afshari et al. 2020	INT	BDI-II: 9.65 (5.82)	BDI-II: 6.42 (3.41)**	YMRS:15.65 (7.19)	YMRS: 10.88 (3.50)**	–	–	DERS:129.80	DERS: 120.07 (17.30)**	–	–
	CTL	BDI-II:10.46 (5.97)	BDI-II: 10.14 (5.81)**	YMRS: 14.39 (7.47)	YMRS: 13.57 (6.96)**	–	–	DERS:136.5 (25.74)	DERS: 135.10 (25.14)**	–	–
Zargar et al. 2019	INT	BDI-II: 20.63 (16.03)	BDI-II: 26.00 (11.63)	YMRS: 4.79 (3.17)*	YMRS: 2.12 (3.09)*/**	–	–	ACS-EC: 172.27 (40.02) ACS-A: 34.12 (10.40) ACS-DM: 36.17(10.12)* ACS-A: 51.78 (14.47) ACS-PA: 49.68 (11.78)	ACS-EC: 162.27 (48.49) ACS-A: 33.00 (11.11) ACS-DM: 31.09 (8.98)*/** ACS-A: 51.30 (16.23) ACS-PA: 47.05 (15.78)	–	–
	CTL	BDI-II: 28.28 (10.76)	BDI-II: 23.76 (11.78)	YMRS: 5.88 (2.91)	YMRS: 4.24 (4.11)**	–	–	ACS-EC: 180.61 (25.44) ACS-Ang: 34.80 (10.39) ACS-DM: 36.12 (8.31) ACS-Anx: 54.67 (11.62) ACS-PA: 54.76 (12.65)	ACS-EC: 188.91 (37.42) ACS-Ang: 37.12 (10.10) ACS-DM: 39.92 (9.05)** ACS-Anx: 56.96 (13.31) ACS-PA: 55.56 (11.88)	–	–
Wright et al. 2020	INT	BDI-II (ITT): 30.30 (11.23)* PHQ-9 (ITT): 17.50 (6.67)*	BDI-II (ITT): 15.60 (10.81)* PHQ-9: 10.00 ( 7.09)*	BMRS (ITT): 2.40 (2.59) ASRM (ITT): 4.40 (4.55)	BMRS (ITT): 1.70 (2.54) ASRM (ITT): 5.30 (2.11)	–	–	–	–	QoL-BD (ITT): 30.00 (6.61)* IIP (PP): 58.44 (18.37)	QoL-BD (ITT): 38.67 (7.86)* IIP (PP): 49.75 (18.27)
Wright et al. 2021	INT	HDRS: 12.8 (8.1) PHQ-9: 15.5 (6.6)	HDRS: 8.5 (4.8) PHQ-9: 10.3 (6.5)	BMRS: 1.8 (2.4)	BMRS: 1.8 (3.2)	–	–	ALS-Total: 37.3 (6.1)	ALS-Total: 26.0(13.5)	QoL-BD: 33.2 (7.5)	QoL-BD: 35.1 (8.3)
	CTL	HDRS: 6.6 (4.2) PHQ-9: 12.9 (5.2)	HDRS: 9.5 (5.8) PHQ-9: 9.8 (4.9)	BMRS: 1.7 (1.8)	BMRS: 1.9 (2.5)	–	–	ALS-Total: 31.5 (13.1)	ALS-Total: 23.5 (12.2)	QoL-BD: 36.6 (6.8)	QoL-BD: 34.0 (10.7)
Valls et al. 2021	INT	HDRS: 6.7 (4.1)	HDRS: 5.0 (3.3)**	YMRS:2.32 (2.0)	YMRS: 2.0 (2.3)	–	–	–	–	**FAST-Total: 23.18 (10.76)** **FAST-C: 4.3 (3.3)** **FAST-L: 2.4 (1.4)** 4/6 FAST domains N.R Qol.BD: 158.36 (29.5)	FAST-Total: 17.1 (9.3)** FAST-C: 2.5 (2.3)** FAST-L: 1.7 (1.28)** 4/6 FAST domains N.R Qol.BD: 154.2 (27.9)
	CTL	HDRS: 3.8 (3.3)	HDRS: 4.5 (4.3)**	YMRS: 1.05 (1.6)	YMRS: 1.22 (2.0)	–	–	–	–	**FAST-Total: 22.95 (14.03)** **FAST-C: N.R** **FAST-L: N.R** 4/6 FAST domains N.R Qol-BD: 160.7 (38.1)	FAST-Total: 23.4 (14.7)** FAST-C: N.R.** FAST-L: N.R.** 4/6 FAST domains N.R Qol-BD: 162.6 (31.59)
Eisner et al. 2017	INT	HDRS: 12.7 (6.0)	HDRS: 11.5 (5.7)	YMRS: 6.1 (6.2)	YMRS: 5.7 (4.0)	–	–	DERS: 107.6 (24.0)* ERS: 52.2 (22.0)*	DERS: 92.8 (23.2)* ERS: 44.1 (21.4)*	–	–
Painter et al. 2019	INT	mHDRS: 3.00 (2.86)	mHDRS: 3.82 (3.87)	YMRS: 2.83 (3.04)	YMRS: 4.6 (5.65)	–	–	ERQ-SE Reappraisal:23.83 (7.92)* ERQ-SE Suppression: 14.67 (4.31)	ERQ-SE Reappraisal: 28.75 (8.13)* ERQ-SE Suppression: 13.50 (6.59)	–	–

^*^ Significant within-group

^**^ Significant between groups

*INT* intervention group*, K-SADS-(DRS/MRS)* kiddie schedule for affective disorders and schizophrenia—(depression rating scale/mania rating scale), *MSSI* modified scale for suicidal ideation*, N.R. not reported, CALS-(C/P)* children’s affective lability scale—(child/parent rating)*, MESSY* matson evaluation of social skills with youngsters,* CTL control group, SIQ-Jr* Suicidal Ideation questionnaire-Junior*, BDI-II* beck depression inventory, *ACS-(EC/Ang/DM/Anx/PA)* affective control scale—(emotional control, anger, depressed mood/anxiety/positive affection), *YMRS* young mania rating scale, *DERS* difficulties in emotion regulation Scale, *PHQ-9* patient health questionnaire 9-item, *BMRS* bech mania rating scale, *ITT* intention-to-treat analysis, *QoL-BD* brief quality of life in bipolar disorder scale, *IIP-32* inventory of interpersonal problems—short version, *PP* per-protocol analysis, *(m)HDRS* (modified) hamilton depression rating scale, *ALS* Affective Lability Scale, *FAST-(C/L)* functioning assessment short test—(cognitive functioning/leisure time), *ERS* emotion reactivity scale, *ERQ-SE* emotion regulation questionnaire-self efficacy

### Child and adolescent studies

Two studies recruited participants with BD younger than 18 years: the same group conducted a small observational study (n = 10) followed by a small RCT (n = 20) in which participants were randomized to DBT or “psychosocial treatment as usual” (psychotherapy primarily consisting of psychoeducational, supportive, and cognitive techniques) (Goldstein et al. 2007, 2015). In both studies, participants were recruited from a specialty mood disorders outpatient clinic and they were already receiving pharmacotherapy. Both studies used the same DBT intervention, which was adapted for the specific population (young, suicidal youths with BD) and alternating between individual therapy sessions and individual-family group skills training over 1 year; DBT team meetings were used in the RCT but not in the observation trial.

Both studies used validated scales measuring depressive symptoms, emotional dysregulation, manic symptoms, suicidality, and interpersonal functioning. The observational study found DBT to be both feasible and acceptable for the adolescent sample. While it also reported statistically significant improvements in depressive symptoms, emotion regulation, and suicidality, there was no significant improvement in manic symptoms, non-suicidal self-injury, or interpersonal functioning. The subsequent RCT confirmed that the intervention was feasible and acceptable, and showed a significantly larger improvement in depressive symptoms in the DBT group than in the control group. However, changes in emotional regulation or suicidality did not differ significantly between the two groups.

### Adult studies

We reviewed eight publications reporting on nine studies that enrolled adult participants with BD: six RCTs and three observational studies. The first published adult study was a wait-list controlled RCT (n = 24) that assessed the feasibility and effectiveness of a DBT skills-based psychoeducational group “(Bipolar Disorders Group(BDG))” for adults with BD in a depressed or euthymic state (Dijk et al. 2013). The BDG consisted of 12 weekly 90-min sessions with eight sessions focused on DBT skills (distress tolerance, emotion regulations, and interpersonal effectiveness skills) and mindfulness skills taught throughout the 12-week intervention. There was only one dropout in each group and attendance and acceptability ratings were similarly high in both. Compared to the wait-list participants, BDG participants demonstrated a significant improvement in mindfulness skills assessed using a mindfulness self-efficacy scale. While they also experienced more improvement in depressive symptoms and in their ability to control emotional states, the difference between the two groups did not reach statistical significance. In the same publication, the authors also reported a significant improvement post-intervention on all symptom measures in a larger sample (n = 75) including their RCT participants and other patients who received the same BDG intervention (Dijk et al. 2013).

Another waitlist-controlled RCT (n = 60) investigated a psychoeducation-focused DBT group intervention based on the BDG of (Dijk et al. 2013, Afshari et al. 2019). Participants were hypomanic, depressed, or euthymic, and were receiving pharmacotherapy. The primary outcome measures selected a priori were executive functioning, mindfulness, and emotion regulation. The intervention group showed significantly more improvement than the control group on the three primary outcome measures and on measures of depression, mania, and emotion dysregulation.

Another RCT compared a 12-week DBT skills intervention and routine pharmacotherapy, with a primary focus on changes in executive functioning, in euthymic adult participants with BD I in maintenance phase (Zargar et al. 2019). While no significant changes were observed in executive functioning or depressive symptoms in either of the two groups, there were significantly higher improvements in manic symptoms and ability to control depressed mood states in the intervention group than in the control group.

A DBT-informed program adapted to target inter-episode mood instability in BD (Therapy for Inter-episode mood Variability in Bipolar (ThrIVe-B)) was assessed in an observational feasibility trial (n = 12) and an RCT (n = 43) (Wright et al. 2020; Wright et al. 2021). Both trials included adult participants with BD who had experienced several subthreshold hypomanic and depressive episodes over the preceding 2 years. Neither study standardized participants’ medications, and 67% and 88% of participants were receiving medications in the pilot and RCT samples, respectively. The 16-week ThrIVe-B program consisted of a combination of group meetings and individual therapy, structured in a modular format with skills-based content (mindfulness, emotion regulation, distress tolerance, and interpersonal effectiveness). The 2020 pilot study established the feasibility and acceptability of the program, based on the recruitment rate, intervention completion rate, and high satisfaction. In addition to its primary aim of confirming feasibility and acceptability, the follow-up RCT evaluated the usefulness of several measures under consideration for use in a future RCT. The RCT confirmed that the ThriVe-B program was feasible and acceptable overall. With respect to effectiveness, the improvements in sense of personal recovery and mindfulness were significantly higher in the intervention group than in the control group, but there were no significant differences between the two groups in the other measures including measures of depression, mania, affective lability, or quality of life.

An RCT compared treatment as usual (TAU), including medication management, with TAU augmented with psychoeducation and several DBT skills (mindfulness, interpersonal effectiveness skills, problem solving) in euthymic adult participants with BD (n = 65) (Valls et al. 2021). There was a significantly larger improvement in the DBT group than in the TAU group on the primary outcome measure, which assessed psychosocial functioning. The improvement in depressive symptoms was similarly significantly higher in the DBT group than in the TAU group; however, baseline depression ratings were low in both groups. There were no significant differences on other measures including mania, anxiety, episode relapse rates, cognition assessed with a 180-min neuropsychological battery, or quality of life.

One observational pilot study investigated a 12-week DBT skills group in euthymic participants with BD I (n = 37) (Eisner et al. 2017). All participants were already treated with pharmacotherapy and at least twice-a-month individual psychotherapy. The group intervention covered skills drawn directly from traditional DBT (mindfulness, emotion regulation, distress tolerance), with a particular focus on emotion regulation. The study demonstrated feasibility and acceptability of the skills group: with 25/37 (68%) participants completing therapy and 22/25 (88%) completers endorsing high satisfaction with the program. Participants also experienced significant improvement in mindfulness, distress tolerance, psychological well-being, post-intervention. There were no significant changes in depressive or manic symptoms; however, the sample had only mild depressive and manic symptoms at baseline.

Finally, an observation study of, a 9-week group-based psychotherapy designed to target emotion dysregulation in BD, has also been published (n = 16) (Painter et al. 2019). All participants had a diagnosis of BD-I and were euthymic, all but one was receiving medications. The program was adapted from an emotion-regulation intervention for patients with psychosis and it used key features of traditional DBT such as didactic training, home practice, and emphasis on DBT skills (emotion regulation, mindfulness, and reappraisal). Overall, the intervention was found to be feasible and acceptable, with 12/16 (75%) participants completing the intervention, 88.0% of sessions attended, and high rates of home practice and participant-rated helpfulness. Participants also demonstrated significant post-intervention improvement in measures associated with wellbeing, including mindfulness, emotion regulation, self-compassion, and affective experiences. There were no significant changes in depressive or manic symptoms.

### Risk of bias

The quality of the reviewed studies is reported in the Additional file 1: Table S1. All studies were evaluated as having a high risk of bias since they were at best single blinded with all participants aware they were receiving the intervention.

## Discussion

We systematically identified and reviewed 10 publications reporting on 11 trials of DBT- informed psychotherapies in participants with BD. These trials assessed interventions based on the traditional DBT protocol (n = 2 trials) (Linehan and Wilks 2015), skills group models adapted for BD (n = 8), or novel therapeutic programs based on core DBT principles and skills (n = 1). Participants in the reviewed studies included both adolescents and adults. When reported, mood states consisted of depressed, hypomanic, or euthymic states with or without inter-episode mood instability; all studies excluded manic mood states. Across the trials, outcomes included depressive or manic symptoms, suicidality, or mindfulness, with some trials focused on other outcomes (e.g., functioning, cognition, or quality of life). Most studies reviewed were pilot and feasibility trials, with only one RCT that was not designed as pilot feasibility studies. All published trials had Ns that were too small (range: 10–108) for reliable conclusions on the efficacy of the assessed intervention. However, these assessed interventions were found to be feasible, with adequate to excellent participant retention and good acceptability reported by participants. Overall, our findings support the need for adequately powered RCTs to assess the efficacy of DBT-based interventions in BD.

In clinical practice, most patients with BD and clinicians favor treatment approaches that combine both pharmacotherapy and psychotherapy. In a recent network and component meta-analysis, manualized psychotherapies were associated with reduced recurrence rates in BD outpatients when compared to control treatments (Miklowitz et al. 2021). Cognitive therapies, which included two DBT studies, were associated with better stabilization of depressive symptoms than TAU. Despite this evidence, psychotherapeutic interventions remain second- and third-line in several clinical guidelines for BD (Yatham et al. 2018). This may be due in part to the relative paucity of published studies in patients with BD compared to the abundance of studies in patients with major depressive disorder or anxiety disorders. To our knowledge, this is the first review synthesizing the evidence related solely to the use of DBT interventions for BD. Beyond establishing feasibility and acceptability, the results were promising with some favorable post-intervention clinical results in all published trials. Despite the limitations of the reviewed studies (see below), the overall evidence suggests potential benefits of DBT for common features of BD such as depressive symptoms, poor emotion regulation, suicidality, or executive dysfunction. Larger and well-designed RCTs are now required.

The major limitations of this review are the small number of relevant studies identified (N = 11). All reviewed trials were limited by their small sample sizes, a high degree of heterogeneity in methods, outcome measures, and participant characteristics, and a high risk for bias. Regardless of their design, all trials were conducted at single sites and were underpowered; future trials may need to be conducted at multiple sites to recruit adequate large samples. About half the trials were observational and the control groups in six RCTs were wait-lists or TAU. Participants were not blinded, which may have influenced reported symptom improvement in both participants and controls. Future RCTs will need to include control conditions that allow for appropriate blinding. All the active interventions included some non-DBT components (e.g., pharmacotherapy, psychoeducation) that may have contributed to any observed clinical improvement; future studies will need to include protocolized pharmacotherapy that control for non-DBT components. The studies assessed DBT for various mood states, contributing to further heterogeneity and making the interpretation of results challenging. All the reviewed studies at least in part recruited patients who were euthymic or exhibiting subthreshold symptoms. Exclusion or omission of participants experiencing clinically significant depressive (or hypomanic) symptoms creates a “floor effect” and may impede the assessment of the symptomatic effect of DBT in patients with BD. Some future studies need to focus on well-characterized subgroups of patients with BD to establish the acute efficacy of DBT for specific mood states, in particular chronic depression which is often the predominant polarity in BD and the most challenging to treat (Baldessarini et al. 2020). Also, all but two of the trials were of short duration (i.e., 16 weeks or less) preventing the assessment of the impact of DBT on the course of BD (e.g., prevention of depressive or manic relapses). Some future studies need to address this issue. Finally, other future studies should build on the available evidence suggesting that DBT can also impact clinically relevant symptoms such as suicidality or emotional dysregulation that are present across mood states and may be less responsive to traditional pharmacotherapy (Dome et al. 2019; Miola et al. 2022).

## Conclusion

Published studies consistently support that DBT-based interventions are feasible and acceptable for patients with BD. These studies also provide some preliminary evidence suggesting these interventions may be effective for improving several core symptoms of BD. Future large and well-designed RCTs are now needed to establish the efficacy of DBT-based interventions for improving specific clinical outcomes in BD.

## Supplementary Information

**Additional file 1**: **Table 1**. Risk of Bias Assessment. **Table 2**. Description of Intervention and Qualitative Outcomes.

## Data Availability

All data generated or analyzed during this study are included in this published article.

## Abbreviations

BD: Bipolar disorders
DBT: Dialectical behavior therapy
FFT: Family focused therapy
CBT: Cognitive behavior therapy
IPSRT: Interpersonal and social rhythm therapy
STEP-BD: Systematic treatment enhancement program for bipolar disorder
TAU: Treatment as usual
BPD: Borderline personality disorder
ICD: International classification of diseases
DSM: Diagnostic and statistical manual of mental disorders
DBT-ST: DBT skills training
MCBT: Mindfulness cognitive behavior therapy
HDRS: Hamilton rating scale for depression
BDI: Beck depression inventory
MADRS: Montgomery–asberg depression rating scale
YMRS: Young mania rating scale
DTS: Distress tolerance scale
MAAS: Mindful attention awareness scale
ThrIVe-B: Therapy for inter-episode mood variability in bipolar
BDG: Bipolar disorder group
