# Supplementary material for: A systematic review on the effectiveness of dialectical behavior therapy for improving mood symptoms in bipolar disorders
Source: Int J Bipolar Disord. 2023 Feb 5;11:6. doi: 10.1186/s40345-023-00288-6 (PMC9899872; doi:10.1186/s40345-023-00288-6)
Supplement: Supplementary file 1 — Additional file 1: Table 1. Risk of Bias Assessment. Table 2. Description of Intervention and Qualitative Outcomes. [file 40345_2023_288_MOESM1_ESM.docx]

Additional file

| Table 1. Risk of Bias Assessment | | | | | | | |
| --- | --- | --- | --- | --- | --- | --- | --- |
| Study | Random Sequence Generation | Allocation Concealment | Blinding (Investigators) | Blinding (Participants) | Selective Outcome Reporting | Missing Data | Overall Bias |
| Goldstein et al., 2007 | N/A | N/A | N/A | N/A | 0 | 1 | High |
| Goldstein et al., 2015 | 0.5 | 0.5 | 0 | 1 | 0 | 1 | High |
| Van Dijk et al., 2013 | 0.5 | 0 | 0.5 | 1 | 0 | 1 | High |
| Afshari et al., 2020 | 0.5 | 0.5 | 0.5 | 1 | 0 | 0 | High |
| Zargar et al., 2019 | 0 | 0 | 0 | 1 | 0 | 0 | High |
| Wright et al. 2020 | N/A | N/A | N/A | N/A | 0 | 0 | High |
| Wright et al., 2021 | 0 | 0 | 0 | 1 | 0 | 0.5 | High |
| Valls et al., 2021 | 0 | 0.5 | 0 | 1 | 0 | 1 | High |
| Eisner et al,, 2017 | N/A | N/A | N/A | N/A | 0 | 0 | High |
| Painter et al., 2019 | N/A | N/A | N/A | N/A | 0 | 0 | High |

0=low risk of bias; 0.5=unclear risk of bias, 1=high risk of bias
Overall bias: low risk of bias (low risk for all domains or some concerns of bias for 1 domain), unclear risk of bias (2 or 3 domains were rated as presenting some cause for concern), and high risk of bias (>3 domains with some bias concerns and/or ≥1domain with high risk of bias).

| **Table 2. Description of Intervention and Qualitative Outcomes** | | | |
| --- | --- | --- | --- |
| **Authors, year** | **Description of intervention** | **Feasibility** | **Acceptability** |
| Goldstein et al., 2007 | *Content*: based on DBT manual by Miller et al. (2006). Standard DBT adapted for adolescent age group and BD diagnosis: less intensive session schedule, incorporation of family members, addition of psychoeducation, and inclusion of BD-tailored DBT skills  *Format*: alternating individual participant therapy sessions and therapist-delivered skills groups with individual family units.  *Duration*: 36 sessions over 12 months  Months 1-6 (acute phase): one 60-minute session per week (24 total), individual vs. group modality alternated weekly  Months 7-12 (continuation phase): 12 total 60-minute sessions, modality alternated monthly.  Optional telephone skills coaching available. | 9/10 participants completed full treatment schedule  Over 90% of all scheduled sessions attended | Frequency of sessions, treatment length, DBT approach and participant gains rated as acceptable and highly satisfactory by parents and participants on post-treatment satisfaction questionnaires |
| Goldstein et al., 2015 | *Content*: Standard DBT adapted for adolescent age group and BD diagnosis: less intensive session schedule, incorporation of family members, addition of psychoeducation, and inclusion of BD-tailored DBT skills  *Format*: alternating individual participant therapy sessions and therapist-delivered skills groups with individual family units.  *Duration*: 36 sessions over 12 months  Months 1-6 (acute phase): one 60-minute session per week (24 total), individual vs. group modality alternated weekly  Months 7-12 (continuation phase): 12 total 60-minute sessions, modality alternated monthly.  Optional telephone skills coaching available (frequency not reported). Optional telephone skills coaching available (frequency not reported) | DBT group attended significantly more therapy sessions than TAU group (p < .01)  Majority (24 of 26) families approached were interested | Parents and participants reported high satisfaction with therapy and participant progress  Non-significant difference on all satisfaction measures in DBT vs. TAU group (all p > .01)  Length of treatment and frequency of visits rated as appropriate in both DBT and TAU groups |
| Van Dijk et al., 2013 | *Content*: DBT-based psychoeducational group named “bipolar disorder group (BDG)”. Intervention had particular focus on mindfulness; traditional DBT skills outlined by Linehan (1993) condensed to fit the adapted shorter duration.  *Format*: single group (n=13)  *Duration*: 12 sessions over 3 months, one 90-min session per week | 12/13 participants completed DBT intervention.  86% of all scheduled sessions were attended | Majority of sample (75%) rated impression of group as “excellent” on evaluation forms |
| Afshari et al., 2020 | *Content*: based on manual by Van Dijk et. Al (2013). Standard DBT adapted for illness (addition of psychoeducation about BD and medications) with a focus on emotion regulation skills.  *Format*: therapist delivered open group (n=8 per group)  *Duration*: 12 sessions over 3 months, one 90-minute session per week | - | - |
| Zargar et al., 2019 | *Content*: intervention taught DBT skills with some session content specific to BD (e.g. disorder facts, causes, medications)  *Format*: therapist delivered groups (n=12-13)  *Duration*: 12 sessions over 3 months, one 1-1.5 hour session per week | - | - |
| Wright et al. 2020 | *Content*: DBT-informed program adapted to target inter-episode mood instability including hypomania, titled “Therapy for Inter-episode Mood Variability in Bipolar Disorder (ThrIVe-B)”. Follows 5 key principles of standard DBT with adapted content for bipolar diagnoses.  *Format*: therapist delivered groups (n=4-8), monthly individual therapy sessions, and custom smartphone app  *Duration*: 16 sessions over 16 weeks, 4 per month.  Additional 30 minute individual sessions, approximately monthly  Follow up ‘booster’ group sessions at 3 and 6 months post intervention | Recruitment rate of 2 participants per month.  9/12 participants (75%) completed treatment  Median 13/16 sessions completed per participant. | 9/10 participants rated “mostly” or “very” satisfied with treatment.  All clinicians rated format and length acceptable and 9/10 rated “very” satisfied with the intervention |
| Wright et al., 2021 | *Content*: same intervention as authors’ 2020 study as above.  *Format*: therapist delivered groups (n=5-9), individual therapy sessions, and custom smartphone app  *Duration*: 18-24 sessions over 15 weeks (18 sessions considered “mandatory” for treatment completion: 15 group sessions, first and last individual sessions, 3-month booster session)  15 closed group sessions once per week.  Up to 8 additional 45-minute individual sessions, approximately monthly  Follow up “booster” group session 3 months post intervention  Midway ‘supporters’ group session offered to include friends/family | Sample total (n=43) below target (n=48)  Recruitment rate 3.9 participants per month  74% participants (32/43) retained at primary end point  50% participants (11/22) completed therapy (defined as completing half of the 18 mandatory sessions), which was below target | 7 of the 8 DBT group participants who provided responses rated treatment as “very” or “extremely” acceptable, and reported being at least “moderately” satisfied with the intervention  Local clinicians (n=8) assessed the DBT as appropriate in length and for the population |
| Valls et al., 2021 | *Content*: integrative intervention combining previously tested components of other treatments effective for BD (psychoeducation, mindfulness, functional remediation).  *Format*: therapist delivered closed groups (n=10-14)  *Duration*: 12 sessions over 3 months, one 90-min session per week | - | - |
| Eisner et al,, 2017 | *Content*: stand alone DBT skills group delivered according to Linehan’s (1993) manual, with focus on residual symptoms and emotion regulation  *Format*: psychologist delivered groups (n=8-11), with skills practice homework  *Duration*: 12 sessions over 12 weeks, one 105-minute session per week  Optional weekly telephone check-in, up to 20-min long | Most of the sample completed treatment (25/37) | 22 participants who completed client satisfaction questionnaire rated intervention positively  88% of completers rated high satisfaction with intervention |
| Painter et al., 2019 | *Content*: skills-based intervention modelled on positive emotion intervention for schizophrenia by Caponigro et al. (2014) titled “Learning Affective Understanding for a Rich Emotional Life (LAUREL)”. 10 skills taught, including BD psychoeducation and skills consistent with DBT (mindfulness, emotion education, reappraisal). Aimed to target emotion dysregulation in BD with a focus on positive emotion regulation  *Format*: therapist delivered group sessions (n=3-6), including skills training, practice, and home practice  *Duration*: 9 sessions over 9 weeks, one 90-min group per week | Majority of participants (12/16) completed treatment with 87.96%.session attendance | High rates of home skills practice (mean 16.37 skills practiced per week)  Participants rated skills as helpful (means ranged 3.00 to 4.11 on 5-point scale) |
| *DBT* dialectical behavioural therapy, *BD* bipolar disorder, *TAU* Treatment as usual, *ThrIVe-B* Therapy for Inter-episode Mood Variability in Bipolar Disorder, *LAUREL* Learning Affective Understanding for a Rich Emotional Life | | | |

**Supplement B**

**Medline Search**

| 1. | exp Bipolar Disorder/ |
| --- | --- |
| 2. | (Bipolar adj3 depress$).mp. |
| 3. | bipolar.mp. |
| 4. | Manic?.mp. |
| 5. | Mania?.mp. |
| 6. | ((Psychosis$ or psychotic$ or psychoses$) adj3 (affective$ or bipolar$ or manic? or mania? or depress$)).mp. |
| 7. | Hypomani$.mp. |
| 8. | Hypermani$.mp. |
| 9. | 1 or 2 or 3 or 4 or 5 or 6 or 7 or 8 |
| 10. | exp Dialectical Behavior Therapy/ |
| 11. | Dialectical behav* therapy.mp. |
| 12. | DBT.mp. |
| 13. | DBT Skills.mp. |
| 14. | exp Mindfulness/ or Mindfulness skills.mp. |
| 15. | distress tolerance skills.mp. |
| 16. | exp Emotional Regulation/ or Emotion regulation skills.mp. |
| 17. | interpersonal effectiveness skills.mp. |
| 18. | 10 or 11 or 12 or 13 or 14 or 15 or 16 or 17 |
| 19. | 9 and 18 |
